# Machine Learning Algorithm to Perform the American Society of Anesthesiologists Physical Status Classification

**DOI:** 10.7759/cureus.47155

**Published:** 2023-10-16

**Authors:** Michael W Lew, Alex Pozhitkov, Lorenzo Rossi, John Raytis, Trilokesh Kidambi

**Affiliations:** 1 Department of Anesthesiology and Perioperative Medicine, City of Hope National Medical Center, Duarte, USA; 2 Division of Research and Informatics, Beckman Research Institute, City of Hope National Medical Center, Duarte, USA; 3 Department of Medicine, Division of Gastroenterology, City of Hope National Medical Center, Duarte, USA

**Keywords:** american society of anaesthesiology physical status (asa ps), asa status, preoperative assessment and risk management, machine learning healthcare data, peri-operative outcomes, oncologic anesthesia

## Abstract

Objective: The American Society of Anesthesiologists (ASA) Physical Status (PS) Classification System defines perioperative patient scores ranging from 1 to 6 (healthy to brain dead, respectively). The scoring is performed and used by physician anesthesiologists and providers to classify surgical patients based on co-morbidities and various clinical characteristics. There is potentially a variability in scoring stemming from individual biases. The biases impact the prediction of operating times, length of stay in the hospital, anesthetic management, and billing. This study's purpose was to develop an automated system to achieve reproducible scoring.

Methods: A machine learning (ML) model was trained on already assigned ASA PS scores of 12,064 patients. The ML algorithm was automatically selected by Wolfram Mathematica (Wolfram Research, Champaign, IL) and tested with retrospective records not used in training. Manual scoring was performed by the anesthesiologist as part of the standard preoperative evaluation. Intraclass correlation coefficient (ICC) in R (version 4.2.2; R Development Core Team, Vienna, Austria) was calculated to assess the consistency of scoring.

Results: An ML model was trained on the data corresponding to 12,064 patients. Logistic regression was chosen automatically, with an accuracy of 70.3±1.0% against the training dataset. The accuracy against 1,999 patients (the test dataset) was 69.6±1.0%. The ICC for the comparison between ML and the anesthesiologists' ASA PS scores was greater than 0.4 (“fair to good”).

Conclusions: We have shown the feasibility of applying ML to assess the ASA PS score within an oncology patient population. Though our accuracy was not very good, we feel that, as more data are mined, a valid foundation for refinement to ML will emerge.

## Introduction

The American Society of Anesthesiologists (ASA) Physical Status (PS) Classification System is used by physician anesthesiologists and providers to classify surgical patients based on co-morbidities and other clinical characteristics. The ASA PS scores range from 1 (healthy) to 6 (brain-dead organ donor) and are assigned by physician anesthesiologists prior to the operative procedure. Originally, the ASA PS score was an assessment of a surgical patient's health status, but now it is used to predict the risk of surgical complications [1]. This practice can result in a patient receiving an ASA PS score, which varies among physician anesthesiologists, which can result in downstream impact as a predictor of operating times, hospital length of stay, postoperative infection rates, necessity of blood transfusion, and overall morbidity and mortality rates [2-6]. Sankar et al. showed moderate inter-rater agreement (kappa=0.61) between the ASA PS scores assigned at a clinic and an operating site for 10,864 patients [7]. In addition, the ASA PS score is used in the determination of anesthesia coding and billing. Because of the utility and the impact of the ASA PS score, it would be beneficial to devise a standardized method for its calculation.

Machine learning (ML) algorithms in artificial intelligence are designed to identify patterns in complex datasets, such as clinical patient data [8,9]. One proposal to improve the ASA PS scoring algorithm is the implementation of ML techniques to refine and automate the computation of the ASA PS score. By utilizing the application of ML in clinical data collection, multiple patient characteristics can be distilled into an objective and consistent ASA PS score. Furthermore, the analysis will extract the relationship between the co-morbidities and the assigned ASA PS scores, which can result in a standard method of assigning ASA PS scores. 

According to the American Cancer Society in the United States, cancer is the second leading cause of death in men and women 45-64 years of age [10]. Considering that oncology is a specialized niche, the physician anesthesiologist's experience, the type of cancer, its stage, and the therapeutic regimens in conjunction with the presence of a coexisting disease(s), the opportunity persists to encounter an even higher variability in the ASA PS score [11,12].

To that point, we propose to develop an ML algorithm that predicts ASA PS scores with a fair degree of confidence for the cancer patient.

This article was previously posted to the medRxiv preprint server on October 7, 2021 (doi: https://doi.org/10.1101/2021.10.05.21264585).

## Materials and methods

City of Hope (COH) is a private, non-profit comprehensive cancer center, and all patients in our database have cancer or are cancer survivors. The study was approved by the COH National Medical Center Institutional Review Board under IRB #17467 "Correlation of Patient Characteristics and Assigned American Society of Anesthesiologists Risk Assessment Scores using a Machine Learning Algorithm." From the 12,064 available patient records, we collected the ASA PS scores produced by the anesthesiologists assigned to the surgical procedures from December 2, 2017, to April 30, 2020. The entire anesthesia department (19 physician anesthesiologists) participated and consisted of a wide range of ages and years in practice. The clinical characteristics, laboratory values, and physician notes were extracted from the COH Enterprise Data Warehouse (COH-EDW), which is a Microsoft SQL database mirroring the data from the Epic electronic medical records system. A custom-made SQL query was created to extract patient records of relevant attributes into a 19095 x 94 matrix. Wolfram Mathematica (Wolfram Research, Champaign, IL) ML was used to automatically select, train, and validate an ML algorithm associating the ASA PS score with clinical characteristics. The analysis was programmed as a multiclass classification problem. ASA PS scores were predicted by the trained model applied to 2,325 patients' records that were not used in training. In a subset of 86 random patients, a physician anesthesiologist (M. Lew) assigned an ASA score based solely on coded diagnosis(es). The anesthesiologist was blinded to the already assigned ASA score determined at the time of surgery. The ASA PS scores were compared to each other. M. Lew’s ASA PS score was compared to 1) the assigned ASA PS score at the time of surgery and 2) the ASA PS score determined by the ML algorithm.

An alternative ML model based on PyCaret (version 2.3.10; PyCaret, Toronto, CA) was developed. The above-mentioned training dataset was transformed by the one-hot encoding of all the diagnosis codes into a 19095 x 4132 matrix. PyCaret then used its default hold-out percentage of 30%, and model training and testing (validation after training) were performed on 13,366 and 5,729 rows of data, respectively. As with Mathematica, the finalized model was tested on 2,325 rows of data not used in training. PyCaret evaluated several classification models, including extra trees, random forest, gradient boosting, light gradient boosting machine, ridge, dummy, logistic regression, linear discriminant analysis, AdaBoost, K-neighbors, decision tree classifier, support vector machines (SVM) - linear kernel, naive Bayes, and quadratic discriminant analysis. Using Wolfram Mathematica, the performance of the models was compared using six commonly used classification metrics: accuracy, AUC, recall, precision, F1, and kappa. This computation with PyCaret was performed on COH's Precision Oncology Software Environment Interoperable Data Ontologies Network (POSEIDON) platform.

## Results

The following variables were extracted from the patient’s records: age at the time of surgery, sex, body mass index, blood pressure systolic and diastolic, pulse rate, respiratory rate, temperature, weight, STOP-Bang score [13], stress test results (specifically if ischemia or reversible ischemia was present), medication of relevant category that was administered within 30 days prior surgery, abnormal laboratory values within 30 days of surgery, and ICD10 codes for diagnoses. Summary statistics of the cohort’s key variables are shown in Table 1.

**Table 1:** Summary of key variable distribution for training and test datasets combined

Variable	Value
Anesthesiologists	19
Cases	21,420
Sex (cases)	
Male (%)	8695 (40.6)
Female (%)	12725 (59.4)
Patients	14063
Age, median (interquartile range)	60.2 (20.1)
ASA 1 (%)	66 (0.8)
ASA 2 (%)	2751 (12.8)
ASA 3 (%)	14176 (66.2)
ASA 4 (%)	4319 (20.2)
ASA 5 (%)	7 (0.005)

The relevant medication categories extracted were opioids, antacids, antianginal, antiarrhythmic, bronchodilator, anticoagulant, anticonvulsant, antidiabetic, antihypertensive, antimyasthenic, antiparkinsonian, antipsychotics/antimanic agents, beta-blockers, calcium blockers, cardiovascular medication, corticosteroids, diuretics, and thyroid medication. If the medication was administered on the same day of surgery, it was labelled as “1” and “0” otherwise.

The abnormal laboratory values extracted were from albumin, alanine transaminase, aspartate aminotransferase, bicarbonate, bilirubin, brain natriuretic peptide, calcium (serum and urine), creatine kinase, creatinine, glucose, hematocrit, hemoglobin, international normalized ratio (INR), prothrombin time, potassium (serum and urine), sodium (serum and urine), platelets, partial thromboplastin time, T3, T4, troponin, thyroid-stimulating hormone, Von Willebrand factor, and white blood cell count. A laboratory value was considered abnormally low/high if, within 30 days prior to surgery, it was measured below or/and above the reference values, respectively. The abnormal laboratory values were labelled as “1.” If the lab test was not done, its value was considered “normal,” because an anesthesia care provider while scoring would not consider a missing test. The normal laboratory values were labelled as “0.” The above-described variables are a significant extension to the list of variables used in a recent ASA PS estimation work done by Zhang et al. [9] and Sobrie et al. [14].

**Figure 1:**
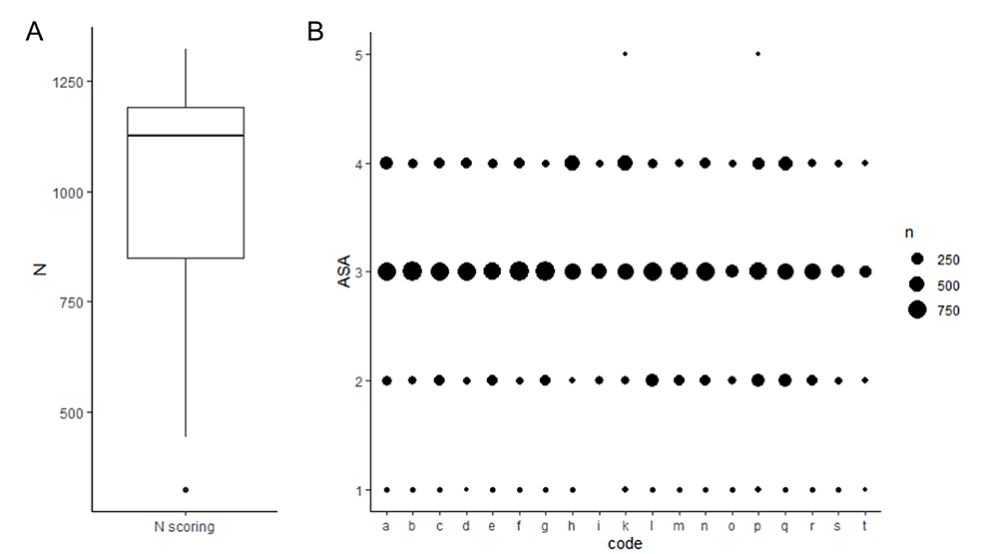
Distribution of the number of scores performed by the 19 physician anesthesiologists (A) and the agreement among the assigned scores (B). Codes “a – t” represent anesthesiologists’ names. Anesthesiologists were randomly assigned to the procedures

Training: An ML algorithm was trained with a dataset comprising 19,095 records corresponding to 12,064 patients (some patients had more than one surgery) from December 2, 2017, to April 30, 2020. Each anesthesiologist was randomly assigned patients and performed preoperative ASA PS scorings (averaging ~1100 cases) in the training dataset. This fact makes the training dataset well covered by all anesthesiologists (Figure 1A), and there is agreement among the providers; however, certain “preferences” may be visually recognized (Figure 1B). Given the substantial number of scorings and overall agreement between the anesthesiologists, we believe the individual biases cancelled each other, and the dataset was suitable for training.

**Figure 2:**
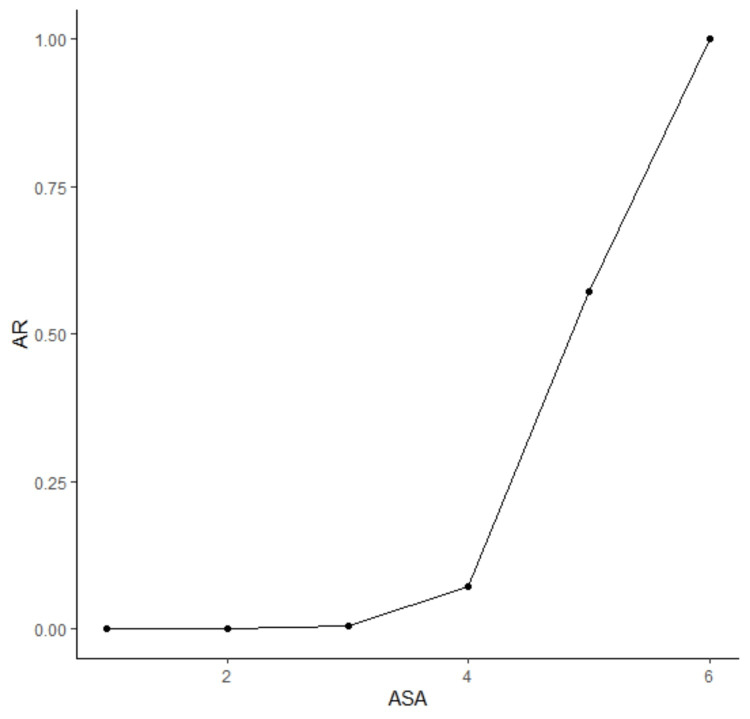
Absolute risk (AR) of dying within 30 days as a function of the ASA physical status class

From the training dataset, we also determined that the absolute risk of dying within 30 days postoperatively was strongly correlated to the ASA score (Spearman rank correlation test, p=0.003; see Figure 2). This finding is consistent with earlier research [4,5] and further emphasizes the need for reproducible ASA PS scoring.

**Figure 3:**
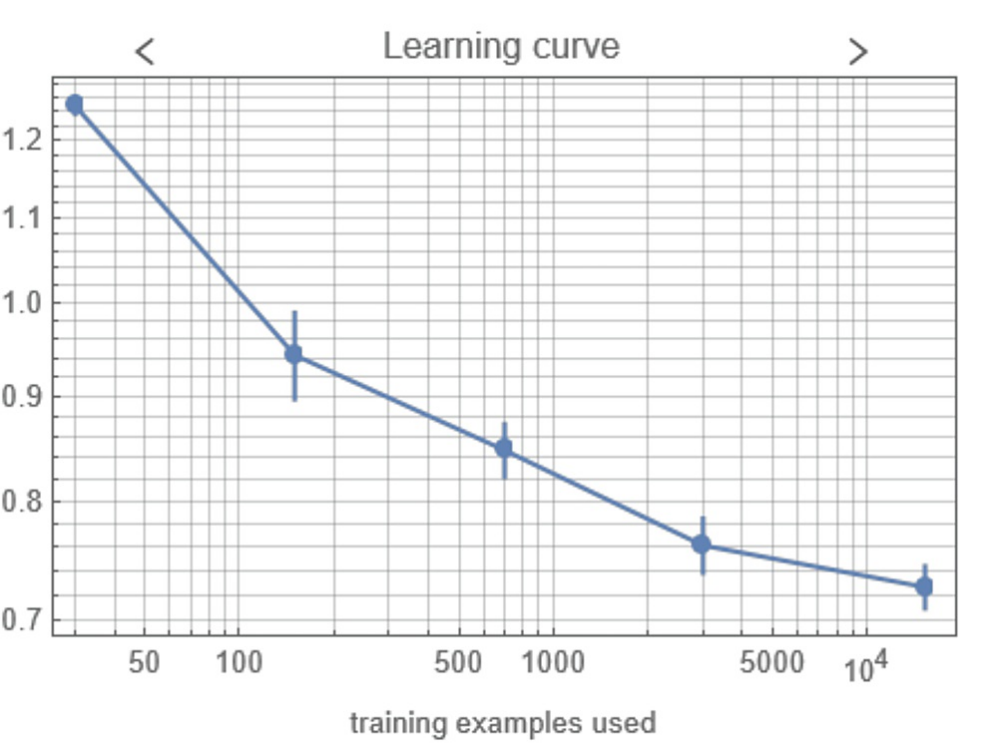
Learning curve (loss vs. number of examples used) for the prediction model training

Wolfram Mathematica automatically determined the appropriate algorithm from the following set: class distributions; decision tree; gradient boosted trees; logistic regression; Markov, naive Bayes, and nearest neighbors' models; neural network; random forest; and support vector machine. The resulting algorithm was logistic regression, and the trained model was exported as a standalone file for re-use. The accuracy of the trained model was 70.3±1.0%. The learning curve is shown in Figure 3.

**Figure 4:**
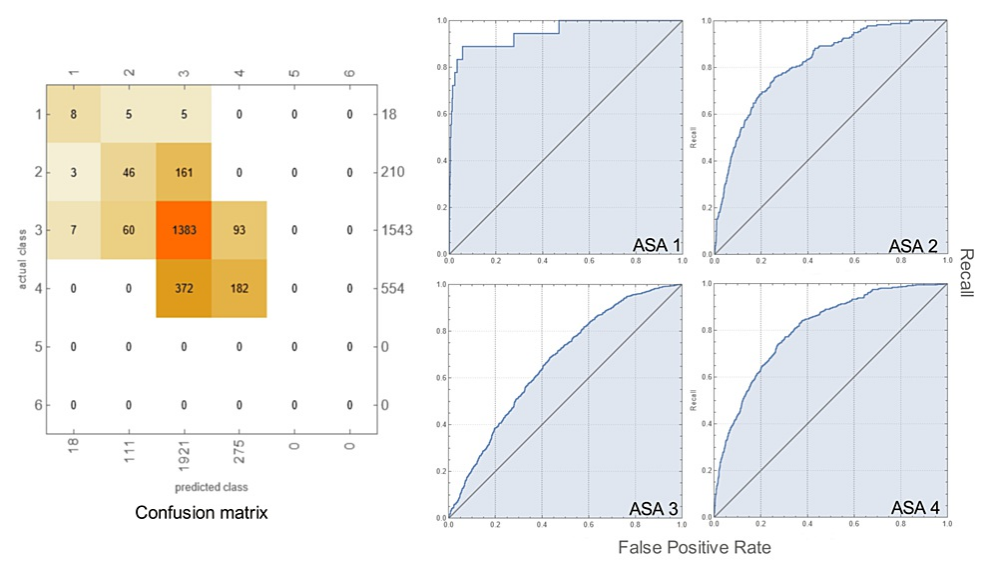
Confusion matrix and ROC curves for the predicted ASA PS class using the trained model

Prediction: The three-month period from Jan 5, 2020, to May 8, 2020, was used to perform a validation experiment; the number of records was 2,325 corresponding to 1,999 patients. Predictions were made by the trained model (above) assigning a case to one of the ASA PS scores. The overall accuracy of predictions was 69.6±1.0%, while the accuracy for the most common score, 3, was 66.4±1.0%. The confusion matrix and the ROC curves are graphically shown in Figure 4.

We took a random subsample of 86 patients and scored them “manually” by M. Lew based on the same attributes that were provided for the training. To assess the concordance between the scorings, the intraclass correlation coefficient (ICC) [15] was calculated for four different comparisons (Table 2). According to Rosner [15], an ICC of less than 0.4 represents poor concordance, while equal or above 0.4 is “fair to good.”

**Table 2:** Intra-class correlation coefficient for comparison between scorings

Comparison	Intraclass confidence interval (ICC)	Note
M. Lew vs. Model	0.303 (0.099-0.483)	86 patients
M. Lew vs. COH	0.334 (0.133-0.509)	86 patients
COH vs. Model	0.243 (0.034-0.431)	86 patients
COH vs. Mode	0.409 (0.374-0.442)	1999 patients

One can see that, as the number of scored patients increases, the concordance between the trained model and the COH anesthesiologists becomes “fair to good.”

The alternative ML model, extra trees, based on the PyCaret platform was selected based on the ranking of the top 5 best performing models. Specifically, the models and respective accuracies were extra trees (70.04%), random forest (69.95%), gradient boosting (69.56%), ridge (68.29%), and light gradient boosting machine (67.66%). We found that the top 10 ranked attributes by their contribution to prediction were as follows: age, body mass index (BMI), weight, systolic blood pressure, pulse, diastolic blood pressure, temperature, respiration rate, low albumin, and low hematocrit. The application of the chosen model to the test dataset (Jan 5, 2020, to May 8, 2020, records) resulted in an accuracy of 70.15 %, which is consistent with the Mathematica results.

## Discussion

Beginning in 1941, the American Society of Anesthesiologists initiated a simple scoring system to assess a patient’s preoperative co-morbidities (Table 3).

**Table 3:** ASA physical status classification

ASA PS Classification	Definition	Adult Examples, Including, but not Limited to:	Pediatric Examples, Including but not Limited to:	Obstetric Examples, Including but not Limited to:
ASA I	A normal healthy patient	Healthy, non-smoking, no or minimal alcohol use	Healthy (no acute or chronic disease), normal BMI percentile for age	
ASA II	A patient with mild systemic disease	Mild diseases only without substantive functional limitations. Current smoker, social alcohol drinker, pregnancy, obesity (30)	Asymptomatic congenital cardiac disease, well-controlled dysrhythmias, asthma without exacerbation, well-controlled epilepsy, non-insulin-dependent diabetes mellitus, abnormal BMI percentile for age, mild/moderate OSA, oncologic state in remission, autism with mild limitations	Normal pregnancy*, well-controlled gestational HTN, controlled preeclampsia without severe features, diet-controlled gestational DM
ASA III	A patient with severe systemic disease	Substantive functional limitations; One or more moderate to severe diseases. Poorly controlled DM or HTN, COPD, morbid obesity (BMI ≥40), active hepatitis, alcohol dependence or abuse, implanted pacemaker, moderate reduction of ejection fraction, ESRD undergoing regularly scheduled dialysis, history (>3 months) of MI, CVA, TIA, or CAD/stents	Uncorrected stable congenital cardiac abnormality, asthma with exacerbation, poorly controlled epilepsy, insulin-dependent diabetes mellitus, morbid obesity, malnutrition, severe OSA, oncologic state, renal failure, muscular dystrophy, cystic fibrosis, history of organ transplantation, brain/spinal cord malformation, symptomatic hydrocephalus, premature infant PCA <60 weeks, autism with severe limitations, metabolic disease, difficult airway, long term parenteral nutrition. Full-term infants <6 weeks of age	Preeclampsia with severe features, gestational DM with complications or high insulin requirements, a thrombophilic disease requiring anticoagulation
ASA IV	A patient with severe systemic disease that is a constant threat to life	Recent (<3 months) MI, CVA, TIA or CAD/stents, ongoing cardiac ischemia or severe valve dysfunction, severe reduction of ejection fraction, shock, sepsis, DIC, ARD or ESRD not undergoing regularly scheduled dialysis	Symptomatic congenital cardiac abnormality, congestive heart failure, active sequelae of prematurity, acute hypoxic-ischemic encephalopathy, shock, sepsis, disseminated intravascular coagulation, automatic implantable cardioverter-defibrillator, ventilator dependence, endocrinopathy, severe trauma, severe respiratory distress, advanced oncologic state	Preeclampsia with severe features complicated by HELLP or other adverse event, peripartum cardiomyopathy with EF <40, uncorrected/decompensated heart disease, acquired or congenital
ASA V	A moribund patient who is not expected to survive without the operation	Ruptured abdominal/thoracic aneurysm, massive trauma, intracranial bleed with mass effect, ischemic bowel in the face of significant cardiac pathology or multiple organ/system dysfunction	Massive trauma, intracranial hemorrhage with mass effect, patient requiring ECMO, respiratory failure or arrest, malignant hypertension, decompensated congestive heart failure, hepatic encephalopathy, ischemic bowel or multiple organ/system dysfunction	Uterine rupture
ASA VI	A declared brain-dead patient whose organs are being removed for donor purposes			

Through the years, the ASA PS scoring system and surgical factors have been used to predict a patient’s perioperative risk [16]. The ASA PS scoring system introduces subjectivity and inter-variability among physician anesthesiologists because of the human interaction (i.e., physical exam, patient feedback) between the physician and the patient during the time of scoring, which is unaccounted for in an ML algorithm. In our study, the goal was to develop and utilize ML algorithm technology to assess multiple data points and ascertain a consistent ASA PS scoring system.

Given that the COH dataset is composed of the scorings of 19 physician anesthesiologists, it is reasonable to believe that the subjectivity was effectively nullified. Hence, the machine was trained on consensus ASA PS scores. Furthermore, one refinement during the process was that ASA PS scores predicted by the algorithm, which varied from the clinician, were reviewed by one anesthesiologist (decreasing practitioner variability) to further improve the consistency and accuracy of the ML model. It is interesting to note that the machine scorings for the data of the three-month period were in fair-to-good agreement with the COH anesthesiologists when all patients were considered. With a small subset of 86 patients manually scored by one chosen independent anesthesiologist, the concordance between the model, the independent anesthesiologist, and COH anesthesiologists’ ASA PS scoring was further away. This finding indicates that over time the ML model will perform as good as the consensus of several anesthesiologists. 

ML models for ASA PS score estimation were developed for general surgical populations in earlier works [9,14] using different supervised classifiers. Our model was trained and evaluated at a specific institution caring solely for oncologic patients. For instance, compared to the cohort presented by Zhang et al. [9], our cohort presented a significantly larger fraction of cases with ASA > 3 (i.e., 20% vs. 8%) and a smaller fraction of cases with ASA < 3 (i.e., 13.6% vs. 48.4%). 

Our study demonstrates the challenges with physician anesthesiologist inter-variability when applying an ML algorithm to ASA PS scoring. One attempt to minimize inter-variability among anesthesiologists and providers is to implement an educational program that was not included in our study. Though it improves consistency, education training alone did not have a statistically significant impact [17]. In addition, ML and artificial intelligence are not immune to bias as humans write and develop the algorithms [18]. Furthermore, our assumption that ML can improve consistency requires further investigation as researchers at Stanford and University of California (UC), Berkeley, recently demonstrated that an artificial intelligence chatbot’s (ChatGPT) accuracy and consistency can fade with time [19]. The challenge is to ethically validate, integrate, and trust artificial intelligence in the clinical anesthesia setting [20,21]. However, the use of ML to delve into large datasets in anesthesia shows promise in morbidity and mortality risk prediction [22].

## Conclusions

The ASA PS scoring process is not a simple set of rules that one could codify and follow, especially as it relates to the oncology patient. As a comprehensive cancer center, we feel confident that the ML algorithm addresses more granular data compared to scoring cancer as one diagnosis, but much work needs to be done. Ultimately a group of “experts” (i.e., physicians, institutions, healthcare networks, and/or payors) can develop qualifying data, train the machine algorithm, and formulate a consistent ASA score. As data collection accumulates and with continual monitoring of the algorithm, this can result in the refinement of the ML ASA scoring algorithm. Our feeling is that ML and artificial intelligence will not replace anesthesiologists but will act as complementary decision-support tools used in shared decision-making with the patient. This is the first known study to apply ML to cancer patients with the goal of minimizing the subjectivity of the ASA score and enhancing patient outcomes.

## References

[1] Sweitzer B (2017). Three wise men (× 2) and the ASA-physical status classification system. Anesthesiology.

[2] Ridgeway S, Wilson J, Charlet A, Kafatos G, Pearson A, Coello R (2005). Infection of the surgical site after arthroplasty of the hip. J Bone Joint Surg Br.

[3] Tang R, Chen HH, Wang YL (2001). Risk factors for surgical site infection after elective resection of the colon and rectum: a single-center prospective study of 2,809 consecutive patients. Ann Surg.

[4] Sauvanet A, Mariette C, Thomas P (2005). Mortality and morbidity after resection for adenocarcinoma of the gastroesophageal junction: predictive factors. J Am Coll Surg.

[5] Prause G, Offner A, Ratzenhofer-Komenda B (1997). Comparison of two preoperative indices to predict perioperative mortality in non-cardiac thoracic surgery. Eur J Cardiothorac Surg.

[6] Carey MS, Victory R, Stitt L (2006). Factors that influence length of stay for in-patient gynecology surgery: is the case mix group (CMG) or type of procedure more important?. J Obstet Gynaecol Can.

[7] Sankar A, Johnson SR, Beattie WS, Tait G, Wijeysundera DN (2014). Reliability of the American Society of Anesthesiologists physical status scale in clinical practice. Br J Anaesth.

[8] Gálvez JA, Jalali A, Ahumada L, Simpao AF, Rehman MA (2017). Neural network classifier for automatic detection of invasive versus noninvasive airway management technique based on respiratory monitoring parameters in a pediatric anesthesia. J Med Syst.

[9] Zhang L, Fabbri D, Lasko TA, Ehrenfeld JM, Wanderer JP (2018). A system for automated determination of perioperative patient acuity. J Med Syst.

[10] (2023). American Cancer Society: Cancer treatment & survivorship facts & figures 2019-2021. https://acsjournals.onlinelibrary.wiley.com/doi/full/10.3322/caac.21565.

[11] Karim HM (2019). American Society of Anesthesiologists physical status: how the obscurity in the system itself contributes to inaccuracies and variations in classification. Korean J Anesthesiol.

[12] De Cassai A, Boscolo A, Tonetti T, Ban I, Ori C (2019). Assignment of ASA-physical status relates to anesthesiologists' experience: a survey-based national-study. Korean J Anesthesiol.

[13] Holt NR, Downey G, Naughton MT (2019). Perioperative considerations in the management of obstructive sleep apnoea. Med J Aust.

[14] Sobrie O, Lazouni ME, Mahmoudi S, Mousseau V, Pirlot M (2016). A new decision support model for preanesthetic evaluation. Comput Methods Programs Biomed.

[15] Rosner B (2006). Fundamentals of Biostatistics, 6th ed. Fundamentals of Biostatistics, 6th ed.

[16] Mayhew D, Mendonca V, Murthy BV (2019). A review of ASA physical status - historical perspectives and modern developments. Anaesthesia.

[17] Knuf KM, Manohar CM, Cummings AK (2020). Addressing inter-rater variability in the ASA-PS classification system. Mil Med.

[18] Nelson GS (2019). Bias in artificial intelligence. N C Med J.

[19] Chen L, Zaharia M, Zou J (2023). How Is ChatGPT’s Behavior Changing Over Time?. https://arxiv.org/pdf/2307.09009.pdf.

[20] Lonsdale H, Jalali A, Gálvez JA, Ahumada LM, Simpao AF (2020). Artificial intelligence in anesthesiology: hype, hope, and hurdles. Anesth Analg.

[21] Hashimoto DA, Witkowski E, Gao L, Meireles O, Rosman G (2020). Artificial intelligence in anesthesiology: current techniques, clinical applications, and limitations. Anesthesiology.

[22] Bellini V, Valente M, Bertorelli G (2022). Machine learning in perioperative medicine: a systematic review. J Anesth Analg Crit Care.

